# Use of 16s rRNA Gene Sequencing for the Identification of Viridans Group Streptococci

**DOI:** 10.7759/cureus.47125

**Published:** 2023-10-16

**Authors:** Thangam Menon, Naveenkumar V

**Affiliations:** 1 Department of Microbiology, Saveetha Institute of Medical and Technical Sciences, Saveetha Dental College and Hospitals, Saveetha University, Chennai, IND; 2 Department of Microbiology, Dr ALM PG Institute of Basic Medical Sciences, University of Madras, Chennai, IND

**Keywords:** gene sequencing, biotyping, 16s rrna pcr, viridans group streptococci, infective endocarditis

## Abstract

Streptococci belonging to the viridans group are gram-positive bacteria residing as commensals in the upper respiratory, gastrointestinal, and genital tracts in humans. Though they are largely known to be commensals, they may also cause life-threatening infections like infective endocarditis, septicemia, pyogenic infections, pneumonia, and meningitis. The viridans group streptococci (VGS) are usually identified by biotyping; however, species discrimination is not always possible by phenotypic characterization. We identified 53 isolates from blood cultures of patients with infective endocarditis and compared the results of biotyping with 16s rRNA gene sequencing for species identification. Organisms belonging to the mitis group were the most common. 16s rRNA gene polymerase chain reaction and sequencing were useful in identifying the etiological agents at the species level.* S.oralis* was the most common etiological agent.

## Introduction

Viridans group streptococci (VGS) are a part of the commensal flora in the upper respiratory, gastrointestinal, and genital tracts in humans [1]. They cause a variety of human infections and are opportunistic pathogens causing diseases, such as infective endocarditis, septicemia, meningitis, and abscesses [2]. Based on phenotypic characteristics, viridans group streptococci are classified into five major groups (sanguinis, mitis, mutans, anginosus, and salivarius) with several species included under each group [3]. Based on the homology of 16S rRNA gene sequences, these bacteria are categorized into four groups (mitis, mutans, anginosus, and salivarius) with members of the sanguinis group included in the mitis group.

The taxonomy of these organisms has been always complex and the classification of VGS has undergone several changes in the recent past with many novel species being added to each group [4,5].

Phenotypic tests are routinely used for speciation of VGS; however, some of the species may be misidentified, because phenotypic traits may often vary among the strains of the same species [3,6]. Compared to biotyping, molecular identification of VGS is considered to be more accurate due to the higher discriminating efficiency at the species level [7]. Accurate identification of these organisms is important for understanding the pathogenic potential of individual species, their association with diseases, and monitoring antimicrobial susceptibility. In this study, we compared biotyping methods with 16S rRNA gene sequencing for the speciation of viridans group streptococci isolates from infective endocarditis.

## Materials and methods

In tota, 53 VGS strains that had been previously isolated from patients with infective endocarditis and maintained in 20% glycerol BHIB broth at -20°C were included in the study. 

Phenotypic characterization of the strains was done using conventional biochemical tests, which included fermentation of mannitol and sorbitol, production of acetoin, hydrolysis of arginine and aesculin, and presence of urease enzyme [3]. 

Molecular characterization was done using 16S rRNA gene sequencing. DNA was extracted by alkaline lysis as described by Hartas et al. [8] and purity was assessed spectrophotometrically using NanoDrop (Thermo Fisher Scientific, Waltham, MA) at 260 and 280 Å. The 16S rRNA gene polymerase chain reaction (PCR) was done using broad-range primers previously described by Weisburg et al [9]. The master mix contained 5 µL of 10× PCR buffer with 15 mM MgCl_2_, 1µL of 10 mM of each dNTP mix, 5 µL of 100 pM of each primer, 0.4 µL of 5 units/µL Taq DNA polymerase, 5 µL of DNA template (0.1-1 µg of DNA) and PCR grade water to make up to a final volume of 50 µL. The PCR was performed in a Mastercycler (Eppendorf, Hamburg, Germany) with initial denaturation for 3 min at 95°C, followed by 37 cycles at 95°C for 30 s, 55°C for 30 s, and 72°C for one min, and a seven min final extension at 72°C. We included positive and negative controls with each run.

The 16S rRNA PCR amplicons were resolved by gel electrophoresis in a 0.8% agarose gel with 1% ethidium bromide for ~30-40 min at 100 V and analyzed by gel documentation (Bio-Rad Laboratories, Hercules, CA).

The 16S rRNA gene PCR was performed in an MJ Research PTC-225 Peltier Thermal Cycler. Sequencing was done using BigDye™Terminator Cycle Sequencing Kits and a 3730XL sequencer (Applied Biosystem, Waltham MA). The sequences obtained were compared with those in the GenBank database using the Basic Local Alignment Search Tool (BLAST). The 16S rRNA gene sequences were deposited in GenBank and accession numbers were obtained.

## Results

Among the 53 VGS strains isolated from IE patients, 39 strains belonged to the mitis group, 10 belonged to the salivarius group, one each belonged to the sanguinis, anginosus, and mutans groups. One strain could not be identified by conventional phenotypic methods. 

Based on 16S rRNA, 52 of the 53 were confirmed to be VGS. The strains were identified as *S. oralis *(27), *S. sangunis* (11), *S. mitis* (1), *S. gordonii* (6), *S. parasanguinus* (3),* S. anginosus* (1), *S. mutans* (1), *S. tigurinus* (1) and* S. cristatus* (1). One strain which was unidentified by biotyping was identified as *S. sanguinis* by 16s rRNA PCR. 

The GenBank Accession numbers were KJ170369- KJ170383, KJ170385-KJ170394, KJ170396, KJ170398-KJ170411, KJ170413- KJ170419, KJ170422-KJ170424, KC007945.1 and GQ411533.

The mitis group members identified by biotyping were speciated as *S. oralis* (27), *S. mitis *(1), *S. tigurinus* (1), and *S. cristatus* (1) by 16S rRNA gene sequencing. All the members in the sanguinis group (S*. sanguinis*,* S. gordonii*,and* S. parasanguinis*) were classified as belonging to the mitis group by 16s rRNA PCR and sequencing; however, only one of them was classified under the sanguinis group by biotyping. Single isolates of *S. anginosus *and *S. mutans* were identified correctly by both methods. Significantly none of the 10 strains identified as salivarius group by phenotyping gave similar results by 16S rRNA gene sequencing (Table 1).

**Table 1:** Comparison of biotyping and 16s rRNA sequencing in the identification of VGS

Groups	No. of Strains in the group (Phenotypic identification)	No identified by 16s rRNA	Species identification by 16s rRNA	No of species
Mitis	39 (73.58%)	50	S.mitis	1 (1.9%)
			S.oralis	27 (51.9%)
			S.sanguinis	11 (21%)
			S.parasanguinis	3 (5.7%)
			S.gordonii	6 (11.5%)
			S.tigurinus	1 (1.9%)
			S.cristatus	1 (1.9%)
Salivarius	10 (18.86%)	0	S.salivarius	0
Mutans	1 (1.8%)	1	S. mutans	1 (1.9%)
Anginosus	1 (1.8%)	1	S.anginosus	1 (1.9%)
Sanguinis	1 (1.8%)	-		
Unidentified	1 (1.8%)	-		
Total	53			52

## Discussion

The viridans group streptococci were formerly referred to as *Streptococcus viridans* because of the greenish color in the blood agar medium around the colony due to the partial lysis of the erythrocytes. This ‘species’ was biochemically diverse and not well defined. Traditionally, biochemical tests such as carbohydrate fermentation, urease production, aesculin and arginine hydrolysis, and acetoin production, have been used to differentiate members in this group [10]. Over the years there have been attempts to standardize these tests and develop commercial identification kits [11], however, a completely satisfactory scheme of identification has not been described.

Over the years, many nucleic acid-based methods have been used to identify and differentiate species of VGS [12]. However, there is no single method that can be used to provide an accurate identification of the species level. Moreover, diagnostic microbiology laboratories are often not equipped to carry out multiple molecular tests. 

We found that 16S rRNA gene sequence analysis-based identification aided in the clear discrimination of most of the members of VGS. It is known that species that are closely related, such as *S. oralis* and *S. mitis*, have significant nucleotide similarities in 16S rRNA sequences and a high level of interspecies recombination, and hence may be misidentified. Only one isolate of *S. sanguinis* was identified as belonging to the sanguinis group by biotyping. Similarly, none of the isolates of *S. parasanguinis* and *S. gordonii* were identified by the phenotypic tests and all the isolates in the salivarius group had been misidentified by biotyping. *Streptococcus oralis* formed more than 50% of our isolates. It is an opportunistic pathogen which resides as a commensal in the oral cavity. It binds to the salivary pellicle and is a key colonizer that helps initiate oral plaque formation. It is also known to produce adhesins and cause platelet aggregation. The mechanisms of causing endocarditis are not well known, but *S. oralis* is known to grow better in thrombotic vegetations when compared to other members of the group [13]. We had one isolate of *S. tigurinus*, a relatively new species belonging to the mitis group which is known to cause IE. This organism was first isolated from the blood culture of a patient with endocarditis in 2012 [14]. Identification of *S. tigurinus* using phenotypic characteristics has been difficult, and commercial identification systems, often identify them as *S. mitis/ S. oralis *because of the limitations in the available database [15]. Hence, *S. tigurinus* is not frequently reported as the etiological agent in invasive infections, particularly in diagnostic laboratories which may rely on biotyping or mass spectrometry for species identification. *S. mutans* is a major cause of dental caries and infective endocarditis; however, we had only a single isolate in our study. 

Species identification of VGS is important for patient management. This study shows that biotyping provides reliable identification only in the case of certain members of VGS who possess prominent differentiable traits. It is not helpful in the identification of some of the species, particularly in the sanguinis and salivarius groups. Hence, the use of phenotypic tests alone may lead to the misidentification of the members of VGS. 16S rRNA gene sequence analysis-based identification is a useful alternative that can be carried out in a diagnostic laboratory, which will serve to provide a more precise identification of the infecting agent. However, there may be limitations in the accurate identification of species that have considerable homology in 16S rRNA gene sequences, particularly *S. oralis* and *S. mitis*.

## Conclusions

The present study shows that though the traditional biotyping scheme is simple to perform and is frequently used for the speciation of VGS, it is able to differentiate only to the group level and does not precisely identify species of some of the groups. 16S rRNA gene sequence analysis-based identification aids in the discrimination between species and could be a better option for the characterization of the members of VGS which have minimal phenotypic differences.
